# MSFANet: a multi-scale feature aggregation network for tobacco pest recognition

**DOI:** 10.3389/fpls.2026.1871587

**Published:** 2026-10-16

**Authors:** Shun He, Sifan Li, Xinjian Li, Qianqian Luo, Sufen Dong, Lu Lei, Xingjiang Chen, Liuti Cai, Jianyu Meng, Ning Lu, Hancheng Wang

**Affiliations:** 1 College of Information Science and Technology, Hebei Agricultural University, Baoding, China; 2 Guizhou Academy of Tobacco Science, Guiyang, China; 3 Qiannan Branch of Guizhou Tobacco Company, Duyun, China

**Keywords:** deep learning, intelligent plant protection, multi-scale feature fusion, real-world images, tobacco pests

## Abstract

**Introduction:**

Tobacco is a globally significant economic crop, and pest infestation represents one of the primary factors contributing to declines in both yield and quality. However, tobacco pests encompass a wide variety of species that differ substantially in morphology and scale. Field-captured images are frequently subject to complex background interference and class imbalance, presenting considerable challenges for automated recognition. Existing deep learning approaches remain limited in their capacity for multi-scale feature extraction and discriminative feature representation under such conditions. To address these challenges, this study proposes a Multi-Scale Feature Aggregation Network (MSFANet) for tobacco pest recognition, formulated here as a single-label image classification task.

**Methods:**

To address scale variation and complex field backgrounds, two task-oriented modules were developed. The Multi-Scale Pyramid Pooling (MSPP) module aggregates contextual information across multiple receptive fields through parallel feature extraction and adaptive cross-branch fusion. The Hierarchical Feature Fusion Block (HFFB) subsequently refines the feature representations through spatial gating and partial convolution. An auxiliary classification head and Focal Loss were incorporated during training to provide intermediate supervision and address class imbalance, respectively.

**Results:**

The effectiveness of MSFANet was evaluated on a dataset comprising 13 tobacco-associated pest and natural-enemy categories. The model achieved accuracy, precision, recall, and F1-score of 97.63%, 97.89%, 97.59%, and 97.71%, respectively, with a parameter count of 1.21 M. Among the models trained from random initialization, MSFANet shared the highest accuracy while using the fewest parameters.

**Discussion:**

By combining multi-scale contextual aggregation with hierarchical feature refinement, MSFANet achieved accurate classification across the 13 tobacco-associated categories under complex field conditions. The proposed method provides effective technical support for automated and precision-oriented pest monitoring in tobacco fields, offering practical value for advancing precise pest management and the broader development of smart agriculture.

## 1 Introduction

Tobacco is one of the most economically significant crops worldwide, cultivated extensively across numerous countries. China alone accounts for approximately 35% of global tobacco production, making it a critical agricultural commodity for the national economy (Liu et al., 2024). Guizhou Province, one of China’s four major tobacco-producing regions, has a cultivated tobacco area of approximately 135, 260 hectares (ha), playing a pivotal role in local economic development (Guizhou Provincial Department of Agriculture and Rural Affairs, 2024). Throughout the growing season, tobacco plants are attacked by diverse sap-feeding, foliage-feeding, and soil-dwelling pests. Their feeding can remove photosynthetic tissue, cause leaf perforation and deformation, and injure seedlings or roots, thereby reducing tobacco yield and the commercial quality of tobacco leaves (Sun et al., 2024; Rabb et al., 1976). Because different pest groups require different management strategies, accurate field classification is essential for timely and targeted pest control. Conventional pest identification methods rely predominantly on manual field surveys, in which trained personnel periodically inspect crops to identify pest species. However, given the vast diversity of pest species, the considerable variation in individual body size, and the morphological similarity among different species, manual identification is inherently time-consuming, labor-intensive, and inefficient, and is further prone to subjective bias (Wang et al., 2020a).

With the rapid advancement of computer vision technology, image recognition-based approaches for the precise identification of crop diseases and pests have emerged as a prominent research direction in this field. Related studies have primarily focused on two methodological categories: traditional machine learning (ML) and deep learning (DL) (Upadhyay et al., 2025). Traditional ML-based methods typically involve preprocessing pest images through operations such as image enhancement, segmentation, and edge detection, followed by the extraction of handcrafted features — including Histogram of Oriented Gradients (HOG), Local Binary Patterns (LBP), and Scale-Invariant Feature Transform (SIFT) — which are subsequently fed into classifiers such as Support Vector Machines (SVM), K-Nearest Neighbors (KNN), and Random Forests (RF) to achieve pest classification (Xiao et al., 2018; Pattnaik and Parvathi, 2022; Kasinathan and Uyyala, 2021). For instance, Ebrahimi et al. (2017) extracted regional indices and color features to detect thrips in strawberry canopy images using an SVM classifier, achieving a mean classification error of less than 2.25%. Faithpraise et al. (2013) applied K-means clustering to segment plant pest images, and subsequently identified different pest types through manually extracted features combined with correspondence filters, enabling automated plant pest detection and recognition. Aravind et al. (2018) extracted bag-of-features and texture descriptors based on statistical histograms and employed SVM for disease classification, attaining a peak recognition accuracy of 83.7%. Nevertheless, these approaches universally rely on extensive handcrafted feature engineering and labor-intensive image preprocessing pipelines, rendering them time-consuming and computationally demanding, with limited generalization capability under complex real-world field conditions.

In contrast, deep learning (DL) methods can learn hierarchical feature representations directly from image data, reducing their dependence on handcrafted feature extraction and preprocessing. Consequently, DL has been increasingly applied to crop disease and pest analysis. For example, Lin et al. (2022a) integrated residual learning and attention mechanisms in GrapeNet to improve grape disease classification, whereas Ye et al. (2023) combined genetic algorithms with stochastic gradient descent to optimize small-pest detection models under field conditions. These studies demonstrate the capacity of DL to extract discriminative visual features from agricultural images and provide a methodological basis for automated pest classification.

The rapid advancement of DL has driven the widespread application of convolutional neural networks (CNNs) in crop disease and pest recognition, with these models demonstrating strong performance across a variety of related tasks (Wang et al., 2025a). Within pest image classification, recent studies have explored hierarchical feature learning, multi-scale feature fusion, transfer learning, multi-task learning, and model ensembles. Lin et al. (2023) proposed the Hierarchical Complementary Network, which integrates fine-grained shallow features with high-level semantic representations and achieved a Top-1 accuracy of 75.3% on the IP102 dataset. Wei et al. (2022) developed a lightweight CNN that combines multi-scale feature fusion with deep semantic representations, obtaining an accuracy of 98.2% across 12 pest categories. Liu et al. (2022a) combined VGG16 and Inception-ResNet-v2 through weighted integration to classify six categories of rice and wheat pests, whereas Dong et al. (2024) introduced a multi-task discriminative-attention framework to improve pest classification on IP102. Nanni et al. (2022) further demonstrated that ensembles of diverse CNN architectures and optimizer variants could improve performance across the Deng, IP102, and Xie2 benchmarks. Collectively, these studies establish the effectiveness of DL for general or crop-specific pest classification. However, because their evaluations were conducted on public benchmarks or non-tobacco-specific pest datasets, they do not directly establish performance for tobacco pest classification under field conditions.

Tobacco-oriented computer vision studies have addressed related but distinct tasks. Lin et al. (2022b) developed CAMFFNet for tobacco disease image classification under field conditions, using multi-depth feature fusion and coordinate attention to reduce feature loss and background interference. Sun et al. (2024) subsequently proposed an enhanced YOLOv8 model for detecting and localizing four common tobacco pest categories in complex environments while reducing model complexity. Although these studies demonstrate the potential of DL for tobacco protection, tobacco disease classification primarily distinguishes symptom patterns expressed on plant tissues, whereas object detection requires bounding-box annotations and jointly predicts pest locations and categories. Neither setting therefore directly evaluates single-label image classification across multiple tobacco pest categories.

Accordingly, evidence remains limited for lightweight, end-to-end image classification across multiple tobacco pest categories under field conditions. In addition to the domain-specific pest composition and imbalanced class distribution, this task requires the model to accommodate variations in pest scale and developmental appearance, distinguish visually similar categories, and suppress interference from complex field backgrounds. To address this gap, this study proposes a Multi-Scale Feature Aggregation Network (MSFANet) for tobacco pest classification. Its Multi-Scale Pyramid Pooling (MSPP) module captures local and contextual information across multiple receptive fields, whereas the Hierarchical Feature Fusion Block (HFFB) refines fine-grained pest features while limiting computational overhead. Focal Loss and an auxiliary classification head are further incorporated during training to address class imbalance and enhance intermediate supervision, respectively. The main contributions of this study are summarized as follows:

1. Designing the MSPP with parallel dilated convolutional branches and multi-scale adaptive pooling branches to simultaneously capture local texture details and multi-granularity contextual information, and introducing a lightweight cross-attention mechanism to adaptively fuse the dual-stream features, enhancing the network’s representational capacity for pest targets across varying scales.
2. Proposing the HFFB, which integrates a Spatial Gating Feature Modulation (SGFM) sub-module for local spatial feature modeling and a Partial Convolutional Feed-forward Network (PCFN) for improved feature representation efficiency, enabling effective feature interaction and fusion under a residual connection framework to strengthen fine-grained pest feature representation in complex field backgrounds.
3. Introducing Focal Loss and an auxiliary classification head as training components, and evaluating their individual and combined effects through controlled ablation experiments.
4. Constructing a dataset comprising 13 tobacco-associated categories and conducting comprehensive experiments to validate the effectiveness of MSFANet, which achieves a classification accuracy of 97.63% with only 1.21 M parameters, demonstrating a favorable balance between classification performance and model complexity.

## 2 Materials and methods

### 2.1 Image acquisition

The 15 principal county-level image-collection areas were distributed across nine prefecture-level administrative regions of Guizhou Province (**Figure 1**; **Table 1**) and ranged in altitude from 628.4 to 2, 115.1 m. Guizhou has a humid subtropical monsoon climate, with a mean annual temperature of approximately 15 °C, long-term mean annual precipitation of approximately 1, 178 mm, and predominantly mountainous and hilly terrain (Han et al., 2024). Yunyan 87 was the predominant cultivar, while K326, Guiyan 20, and other cultivars were grown at smaller scales.

**Figure 1:**
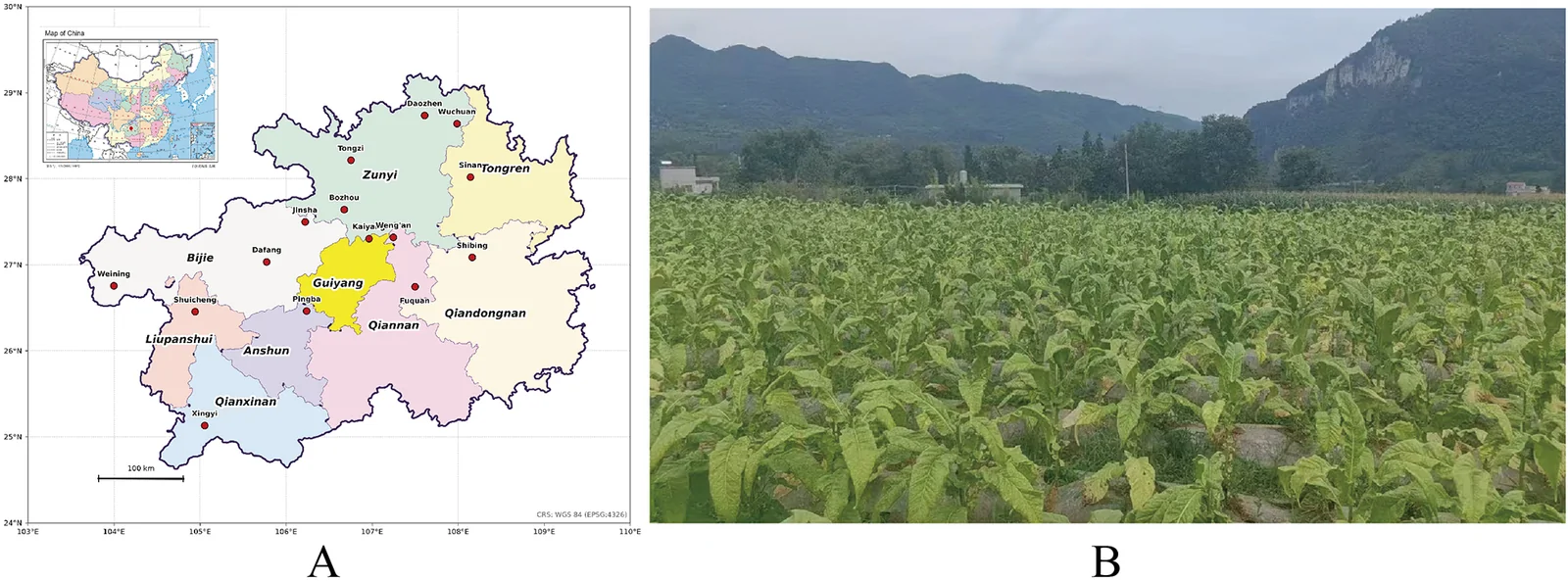
Image acquisition area. **(A)** Geographic distribution of the 15 principal county-level image-collection areas across Guizhou Province, China. Red dots indicate tobacco pest image-collection sites, and the inset shows the location of Guizhou Province within China. Adapted from Standard Map Service of Ministry of Natural Resources of China. Citation: GS(2016)1600号; Source: http://bzdt.ch.mnr.gov.cn/; License: Free for academic use with attribution. **(B)** Representative tobacco-field scene.

**Table 1:** Geographic information for the 15 image collection sites in Guizhou Province, China.

Prefecture-level region	Image collection site	Coordinates (WGS 84)	Altitude (m)
Zunyi	Wuchuan Gelao and Miao Autonomous County	28.639531° N, 107.986831° E	628.4
Zunyi	Bozhou District	27.640789° N, 106.676031° E	1215.7
Zunyi	Tongzi County	28.214050° N, 106.752136° E	1100.0
Zunyi	Daozhen Gelao and Miao Autonomous County	28.734275° N, 107.608947° E	1319.9
Tongren	Sinan County	28.017919° N, 108.142731° E	969.4
Qiandongnan Miao and Dong Autonomous Prefecture	Shibing County	27.085000° N, 108.163692° E	800.3
Qiannan Buyei and Miao Autonomous Prefecture	Fuquan City	26.745697° N, 107.497389° E	1010.8
Qiannan Buyei and Miao Autonomous Prefecture	Weng’an County	27.317850° N, 107.246819° E	968.1
Guiyang	Kaiyang County	27.303458° N, 106.961908° E	860.7
Anshun	Pingba District	26.461019° N, 106.238944° E	1317.7
Qianxinan Buyei and Miao Autonomous Prefecture	Xingyi City	25.131047° N, 105.052956° E	1272.7
Liupanshui	Shuicheng District	26.453528° N, 104.942950° E	1790.7
Bijie	Weining Yi, Hui and Miao Autonomous County	26.753564° N, 103.996586° E	2115.1
Bijie	Dafang County	27.032369° N, 105.774200° E	1278.0
Bijie	Jinsha County	27.496647° N, 106.219906° E	938.9

Coordinates represent the administrative centres of the corresponding county-level collection areas rather than the exact locations of individual tobacco fields; altitudes refer to the sampled tobacco fields.

The dataset comprised 10, 968 images representing 12 pest categories and one natural-enemy category. The images represented adult, larval, or juvenile stages, as applicable to each category. The category-wise image counts were 364 for green rice bug (*Nezara viridula*), 847 for cutworm (*Agrotis* spp.), 1, 552 for locust (*Locusta migratoria*), 766 for scarab beetle (*Anomala corpulenta*), 1, 035 for slug (*Deroceras reticulatum*), 400 for mole cricket (*Gryllotalpa orientalis*), 527 for hoverfly (*Episyrphus balteatus*), 968 for snail (*Achatina fulica*), 787 for tobacco cutworm (*Spodoptera litura*), 787 for tobacco budworm (*Helicoverpa assulta*), 1, 041 for aphid (*Myzus persicae*), 1, 039 for long-winged tree cricket (*Oecanthus longicauda*), and 855 for katydid (*Tettigonia viridissima*). **Figure 1** also presents a representative tobacco-field scene, and representative images from each category are shown in **Figure 2**.

**Figure 2:**
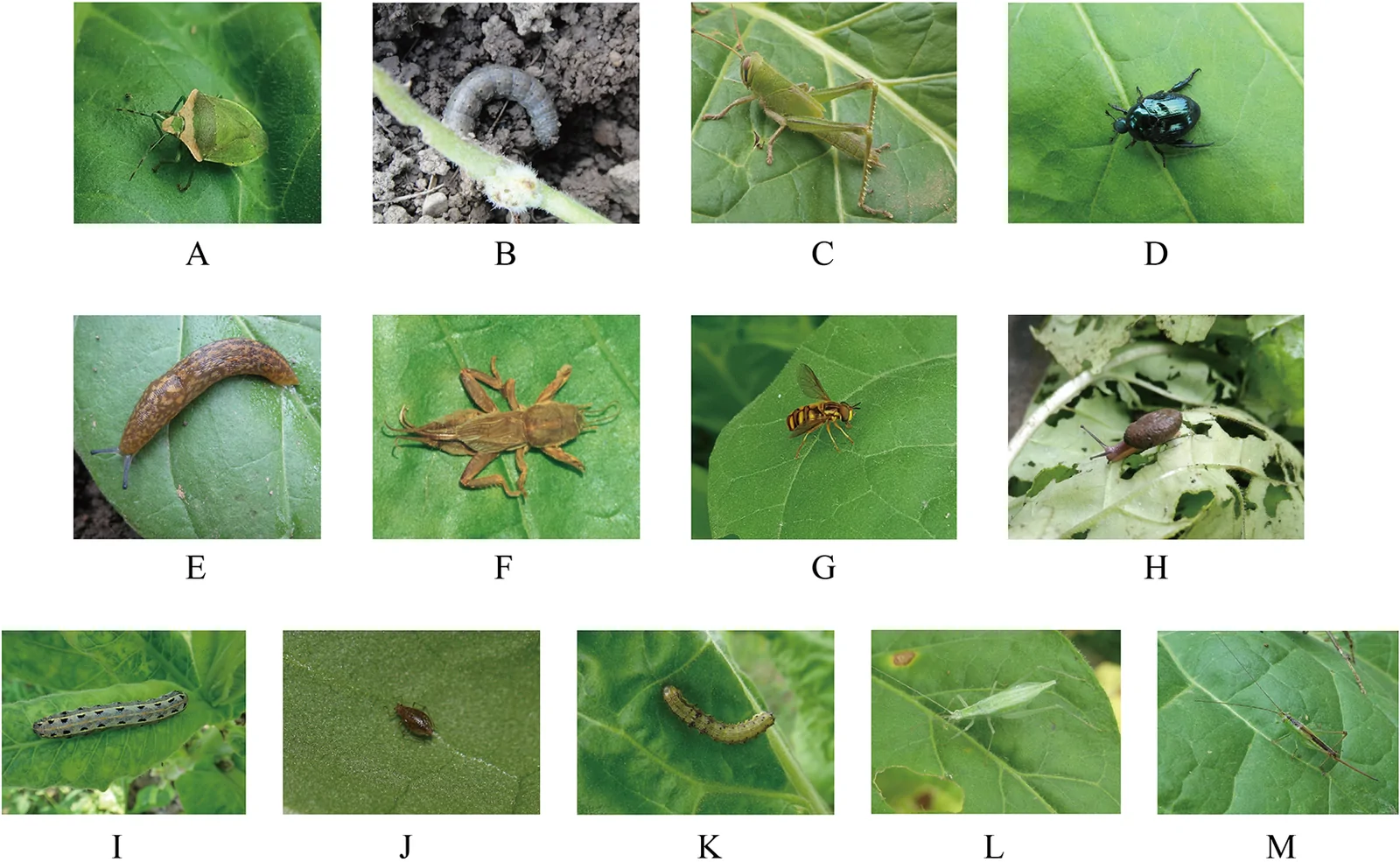
Representative images of the tobacco-associated categories: **(A)** green rice bug. **(B)** cutworm. **(C)** locust. **(D)** scarab beetle. **(E)** slug. **(F)** mole cricket. **(G)** hoverfly. **(H)** snail. **(I)** tobacco cutworm. **(J)** aphid. **(K)** tobacco budworm. **(L)** long-winged tree cricket. **(M)** katydid.

### 2.2 Image preprocessing

A total of 10, 968 images were acquired under field conditions by multiple collectors using different smartphones and digital cameras, with device models and acquisition settings varying across images. All images were individually reviewed by plant protection experts to verify image quality and class labels. Each image was assigned one of 13 predefined category labels. Most images contained a single individual, whereas some contained multiple individuals belonging to the same category; no image contained individuals from different categories. Accordingly, each image constituted one sample in the single-label classification task, irrespective of the number of individuals present. Images from each category were stratified into training, validation, and test sets at an 8:1:1 ratio, with the resulting distribution reported in **Table 2**.

**Table 2:** Distribution of image data by category.

Category	Train	Valid	Test
green rice bug	292	36	36
cutworm	678	84	85
locust	1242	155	155
scarab beetle	613	76	77
slug	828	103	104
mole cricket	320	40	40
hoverfly	422	52	53
snail	775	96	97
tobacco cutworm	630	78	79
aphid	833	104	104
tobacco budworm	631	78	78
long-winged tree cricket	832	103	104
katydid	684	85	86
Total	8780	1090	1098

Deep learning models typically rely on data augmentation techniques to increase the diversity of training samples, thereby improving model accuracy, generalization ability, and robustness, while effectively alleviating overfitting (Jia et al., 2017). To improve robustness to variations in pest scale, orientation, illumination, color, and partial occlusion, the training images were resized to 252 × 252 pixels and randomly cropped to 224 × 224 pixels using a scale range of 0.88–1.00 and an aspect-ratio range of 0.75–1.33. Random horizontal and vertical flips were applied with probabilities of 0.50 and 0.25, respectively, together with random rotations within ±12°. Color jitter was applied to brightness, contrast, and saturation using a factor of 0.12 and to hue using a factor of 0.06. Random erasing was applied with a probability of 0.20, an area range of 0.02–0.12, and an aspect-ratio range of 0.30–3.30. Images were subsequently converted to tensors and normalized using ImageNet means of (0.485, 0.456, 0.406) and standard deviations of (0.229, 0.224, 0.225). Validation and test images were resized directly to 224 × 224 pixels and normalized identically, without stochastic augmentation. All stochastic transformations were applied online during data loading rather than used to generate a fixed augmented copy of the dataset. Therefore, no fixed post-augmentation dataset size existed.

## 3 Proposed method

### 3.1 Overall architecture of MSFANet

Tobacco pests encompass a wide variety of species that differ substantially in morphology and scale across individual specimens. Moreover, field-captured images are frequently subject to interference from varying illumination conditions, cluttered backgrounds, and partial occlusion. To address these challenges, this study proposes MSFANet, whose overall architecture is illustrated in **Figure 3**. The network consists of three principal components: a Stem module, a multi-scale feature aggregation backbone, and a channel-spatial attention module.

**Figure 3:**
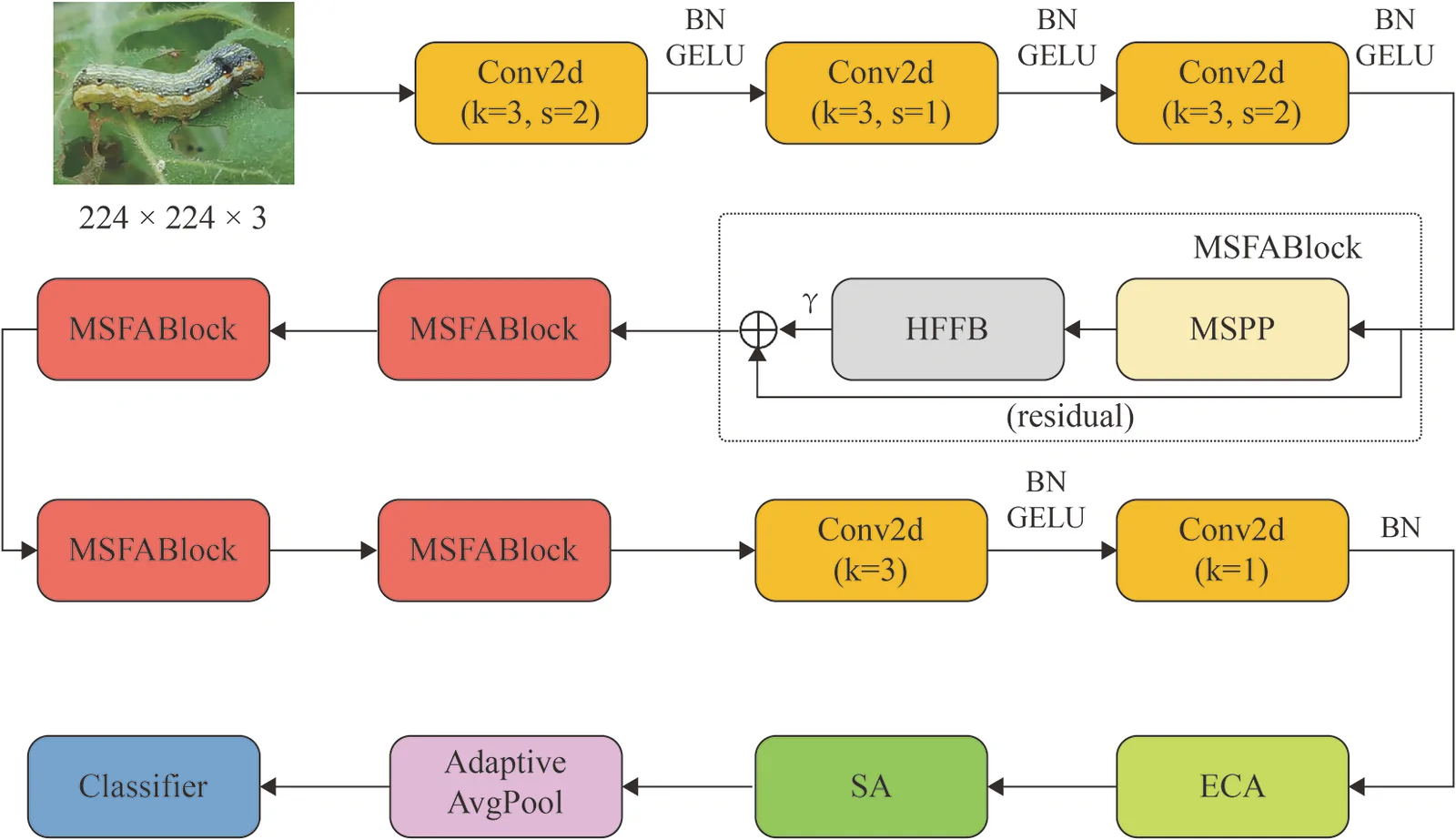
MSFANet architecture.

Within this architecture, the stem transforms an input image of 3 × 224 × 224 into an 80 × 56 × 56 feature representation through three consecutive 3 × 3 convolutions, two of which use a stride of 2. The resulting representation is subsequently processed by five sequential MSFA blocks, each comprising MSPP, two-dimensional dropout (Dropout2d; rate = 0.15), and HFFB. Each block also incorporates a learnable residual scaling factor γ, initialized to $10^{-6}$. Because no further strided operations are applied, the backbone maintains a feature dimension of 80 × 56 × 56 throughout the five blocks. The subsequent feature-enhancement module increases the channel dimension from 80 to 160 while preserving the spatial resolution. Global average pooling and the main classifier then map the enhanced representation to the 13 tobacco-associated categories. During training, an auxiliary classifier branches from the output of the third MSFA block to provide intermediate supervision. The layer-wise configuration of MSFANet is summarized in **Table 3**.

**Table 3:** Configuration of the MSFANet model.

Layer name	Input shape	Output shape	Configuration
Input	–	3 × 224 × 224	–
3 × 3 Conv	3 × 224 × 224	40 × 112 × 112	Conv(s = 2, p = 1), BN, GELU
3 × 3 Conv	40 × 112 × 112	40 × 112 × 112	Conv(s = 1, p = 1), BN, GELU
3 × 3 Conv	40 × 112 × 112	80 × 56 × 56	Conv(s = 2, p = 1), BN, GELU
MSFA Block 1	80 × 56 × 56	80 × 56 × 56	MSPP → HFFB; DropPath = 0
MSFA Block 2	80 × 56 × 56	80 × 56 × 56	MSPP → HFFB; DropPath = 0.0375
MSFA Block 3	80 × 56 × 56	80 × 56 × 56	MSPP → HFFB; DropPath = 0.075
Auxiliary classifier¹	80 × 56 × 56	13	BN, GAP, Flatten, Dropout (0.15), FC (80→13)
MSFA Block 4	80 × 56 × 56	80 × 56 × 56	MSPP → HFFB; DropPath = 0.1125
MSFA Block 5	80 × 56 × 56	80 × 56 × 56	MSPP → HFFB; DropPath = 0.15
3 × 3 Conv	80 × 56 × 56	160 × 56 × 56	Conv(s = 1, p = 1), BN, GELU
1 × 1 Conv, ECA, SA	160 × 56 × 56	160 × 56 × 56	Conv(s = 1), BN, ECA, SA
Global pooling	160 × 56 × 56	160	BN, GAP, Flatten
Main classifier	160	13	Dropout (0.30), FC (160→80), GELU, Dropout (0.15), FC (80→13)

¹The auxiliary classifier branches from MSFA Block 3 and is used only during training.

The main and auxiliary classification outputs are jointly optimized using Focal Loss, and the resulting training objective is defined as Equation 1:

(1) $$L_{total}=L_{main}+\lambda \cdot L_{aux}$$

where $L_{\text{main}}$ and $L_{\text{aux}}$ denote the Focal Loss outputs of the primary classification head and the auxiliary classification head, respectively, and λ represents the balancing coefficient for the auxiliary loss. The auxiliary-loss weight λ was fixed at 0.30 throughout training, and no decay schedule was applied.

### 3.2 Multi-scale pyramid pooling

Convolutional operations with a single receptive field are inherently limited in their ability to simultaneously capture both fine-grained textural details of small-bodied pests and the macroscopic morphological characteristics of large-bodied species. Existing methods typically employ fixed-scale convolutional kernels for feature extraction, which are ill-suited to the substantial target scale variation present in tobacco pest images (Yang et al., 2025). To address this limitation, this study designs the MSPP module, whose architecture is illustrated in **Figure 4**. MSPP comprises four synergistic components: a dilated convolutional branch, a pooling branch, a global average pooling branch, and a lightweight cross-attention fusion mechanism. Following the final feature fusion, the aggregated representations are sequentially refined through Efficient Channel Attention (ECA) and Spatial Attention (SA) for dual-stage feature enhancement, enabling the network to extract discriminative cross-scale feature representations under complex field backgrounds.

**Figure 4:**
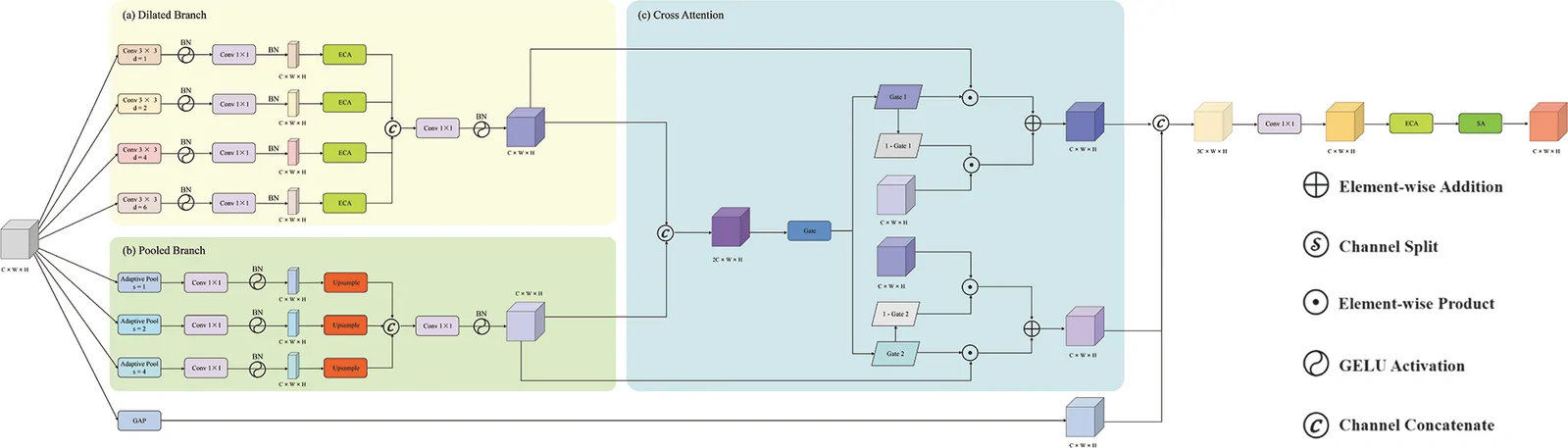
Structure of multi-scale pyramid pooling.

The dilated convolutional branch, as shown in **Figure 4a**, consists of four parallel sub-branches with dilation rates d∈{1,2,4,6}. Here, $X\in R^{B\times C\times H\times W}$ denotes the input feature map of MSPP, where B, C, H, and W represent the batch size, channels, height, and width, respectively. It is produced by the stem for the first MSFA block and by the preceding MSFA block thereafter; throughout the backbone, C=80 and H=W=56. Dilated convolution effectively enlarges the receptive field without increasing the number of parameters (Chen et al., 2018; Hu et al., 2024), enabling the model to simultaneously capture fine-grained textural features of small-bodied pests (e.g., aphids) and macroscopic morphological characteristics of large-bodied species (e.g., locusts). Within each sub-branch, the feature extraction pipeline consists of depthwise separable convolution, Batch Normalization (BN), GELU activation, 1×1 pointwise convolution, BN, and ECA channel attention applied in sequence. The feature extraction process of the d-th sub-branch is formulated as Equation 2:

(2) $$F_{d}=ECA(BN(Conv_{1\times 1}(GELU(BN(DWConv_{3\times 3}^{d}(X)))))),\;d\in \{1,2,4,6\}$$

where $DWConv_{3\times 3}^{d}$ denotes the 3×3 depthwise separable convolution with dilation rate d, $Conv_{1\times 1}$ denotes pointwise convolution, *BN* denotes batch normalization, and *GELU* denotes the activation function. The output features of the four sub-branches are concatenated along the channel dimension and subsequently compressed to *C* channels via a 1×1 convolutional layer, yielding the fused feature representation of the dilated convolutional branch.

The pooling branch, illustrated in **Figure 4b**, consists of three parallel adaptive average pooling sub-branches operating at pooling scales s∈{1,2,4}. By performing spatial compression at varying degrees, this branch is capable of capturing multi-scale regional semantic information ranging from fine-grained to coarse-grained representations, which facilitates the representation of pest-relevant regions against complex field backgrounds (Wang et al., 2024b). Within each sub-branch, the spatial resolution is first reduced via adaptive average pooling, followed sequentially by 1×1 convolution, BN, and GELU activation. The resulting feature maps are then restored to the original spatial resolution through bilinear interpolation upsampling. The output of the s-th sub-branch is formulated as Equation 3:

(3) $$P_{s}=\text{Upsample}(\text{GELU}(\text{BN}({\text{Conv}}_{1\times 1}({\text{AdaPool}}_{s}(X))))),\;s\in \{\mathrm{1; 2; 4}\}$$

The output features of the three sub-branches are concatenated along the channel dimension and subsequently compressed via a 1×1 convolutional layer, yielding the fused feature representation of the pooling branch, denoted as $F_{pool}$. Additionally, a global average pooling branch compresses the input feature map into a global semantic vector, which is then spatially expanded to C×H×W through a 1×1 convolutional layer, producing the global contextual feature $F_{ɡap}$ to provide image-level semantic information for subsequent feature fusion.

The dilated convolutional branch focuses on capturing local multi-scale texture information, while the pooling branch specializes in extracting regional semantic information; the two branches are thus naturally complementary to one another. However, simple static fusion strategies such as element-wise addition or channel concatenation are unable to adaptively adjust the contribution weights of each branch according to the semantic differences across spatial positions. To this end, a lightweight Cross-Attention mechanism is introduced to achieve dynamic and mutually complementary fusion of the two-branch features, as illustrated in **Figure 4c**. Specifically, $F_{dil}$ and $F_{pool}$, each with dimensions C×H×W, are concatenated along the channel dimension to obtain a 2C×H×W representation. The gating network comprises two bias-free 1 × 1 convolutions. The first convolution reduces the channel dimension from 2C to ${\text{C}}_{\text{g}}=\text{ max}(\text{C}/4,\;16)$, followed by BN and GELU, whereas the second restores the channel dimension to 2C and applies sigmoid activation. For C=80, this bottleneck implements a channel mapping of 160→20→160. The resulting representation is divided into two C-channel gating maps, $G_{1}$ and $G_{2}$, which perform complementary modulation of the two feature streams as expressed in Equations 4, 5:

(4) $${\hat{F}}_{dil}=F_{dil}⊙G_{1}+F_{pool}⊙(1-G_{1})$$

(5) $${\hat{F}}_{pool}=F_{pool}⊙G_{2}+F_{dil}⊙(1-G_{2})$$

where ⊙ denotes element-wise multiplication, and $G_{1}$, $G_{2}$ represent the spatially adaptive gating weights. This dynamic gating design enables the network to adaptively suppress background interference and highlight target region features according to the semantic response intensity at each spatial position, making it better suited for tobacco pest classification under complex field conditions. Finally, the three feature representations ${\hat{F}}_{dil}$, ${\hat{F}}_{pool}$, and $F_{ɡap}$ are concatenated along the channel dimension and sequentially refined through ECA and SA modules to produce the final output features of MSPP. By maintaining the input spatial resolution and channel dimensionality, MSPP effectively integrates multi-scale contextual semantics and cross-branch complementary information, providing high-quality input representations for the subsequent hierarchical feature refinement performed by the HFFB.

### 3.3 Hierarchical feature fusion block

Following multi-scale feature aggregation by the MSPP module, the resulting feature representations require further hierarchical refinement to enhance the network’s discriminative capacity for subtle morphological differences among pest species. To this end, this study designs a HFFB, whose structure is illustrated in **Figure 5**. The HFFB consists of a Spatial Gating Feature Modulation (SGFM) module and a Partial Convolutional Feed-forward Network (PCFN) connected in series, both of which adopt residual connections, forming a hierarchical feature enhancement mechanism following the paradigm of “spatial modulation — partial mapping — residual fusion”. This design enables the network to effectively capture subtle textural variations and local morphological characteristics in tobacco pest images.

**Figure 5:**
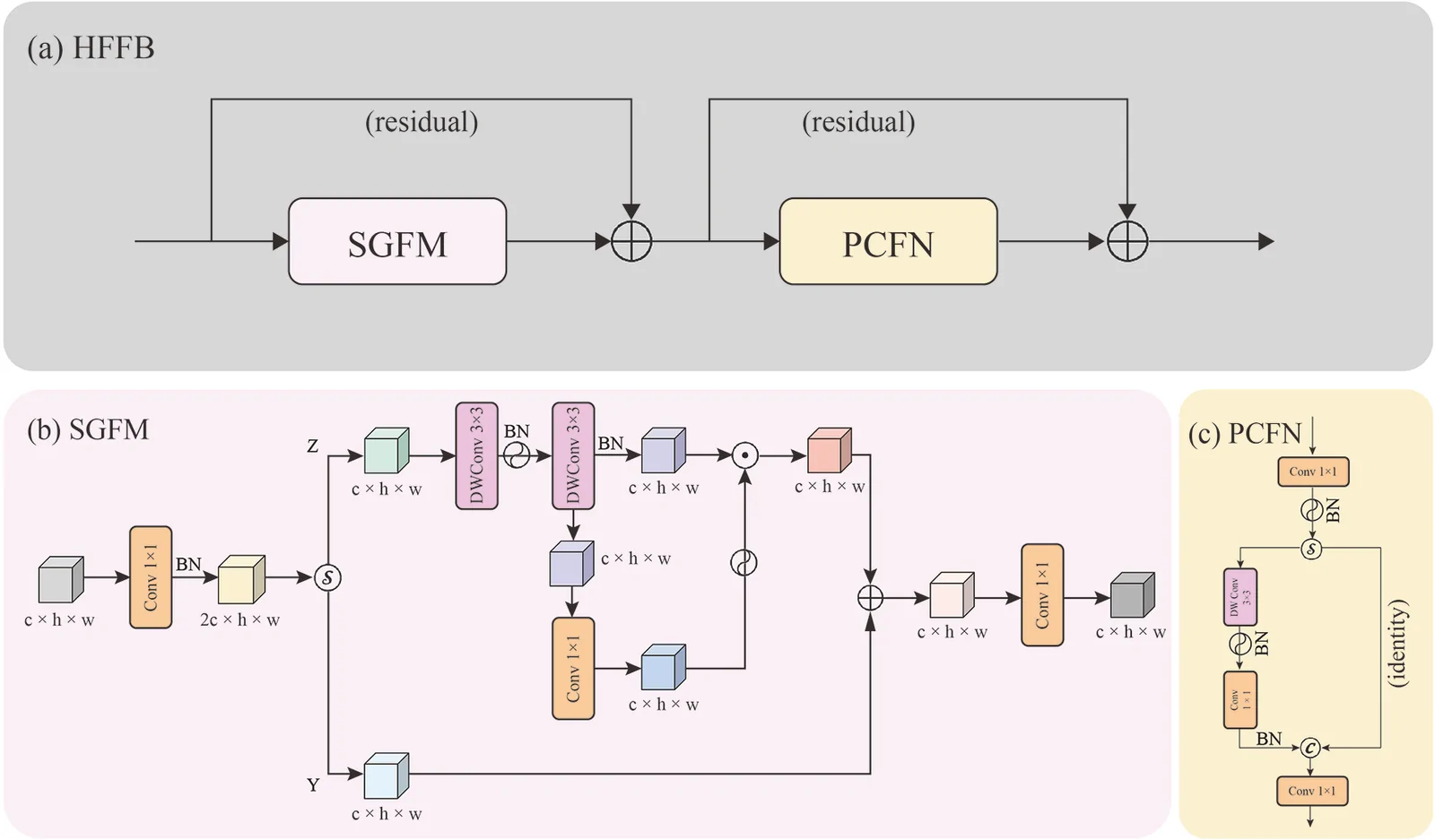
Structure of the hierarchical feature fusion block (HFFB). **(a)** Overall architecture of the HFFB. **(b)** Detailed structure of the SGFM. **(c)** Detailed structure of the PCFN.

The structure of SGFM is illustrated in **Figure 5b**. Conventional feed-forward feature transformations typically process all spatial positions uniformly, making it difficult to adaptively focus on the key discriminative regions of pest targets (Wang et al., 2025b). SGFM addresses this limitation by performing adaptive information filtering and modulation of feature maps through a spatial gating mechanism. Specifically, the channel dimensionality of the input features is first expanded to 2C, after which the expanded features are evenly split along the channel dimension into two streams, Y and Z. Here, Y is retained as a bypass branch, whereas Z is processed by two sequential 3 ×3 depthwise convolutions to obtain $Z_{dw}$. The resulting feature is processed by a 1×1 convolution followed by GELU to generate the gating response, as formulated in Equation 6:

(6) $$\begin{array}{c} {\text{Z}}_{\text{dw}}=\text{BN}({\text{DConv}}_{3\times 3}(\text{GELU}(\text{BN}({\text{DConv}}_{3\times 3}(\text{Z}))))),\;\\ {\text{Z}}_{gate}=Z_{dw}⊙GELU(Conv_{1\times 1}(Z_{dw})) \end{array}$$

The gating-modulated feature ${\text{Z}}_{ɡate}$ is then added to the gating branch Y and subsequently projected through a 1×1 convolutional layer to complete channel mapping, yielding the output of SGFM as formulated in Equation 7. This gating design enables the network to dynamically highlight pest target region features according to the response intensity at each spatial position, effectively suppressing interference from complex field backgrounds.

(7) $${\text{F}}_{SGFM}=Conv_{1\times 1}(Z_{ɡate}+Y)$$

Inspired by the concept of partial convolution (Liu et al., 2023), this study designs a PCFN, as illustrated in **Figure 5c**. Unlike standard feed-forward networks that apply convolutional transformations to all channels, PCFN processes only a subset of the expanded features. Specifically, the 80-channel input is expanded to 212 channels using an expansion factor of 2.66. With the partial-convolution ratio set to ${\text{C}}_{\text{p}}\;=\;0.5$, 106 channels are processed by the partial pathway, which comprises a 3 × 3 depthwise convolution followed by a 1 × 1 pointwise convolution, while the remaining 106 channels bypass this operation. The two feature subsets are subsequently concatenated and projected back to 80 channels, as formulated in Equation 8:

(8) $${\text{F}}_{PCFN}=Conv_{1\times 1}([F_{p}^{conv};{\text{F}}_{r}])$$

where $F_{p}^{conv}$ denotes the features of the first $C_{p}$ channels processed through the partial convolutional pathway, ${\text{F}}_{r}$ denotes the directly preserved residual channel features, and [·;·] denotes channel-wise concatenation. This selective convolution strategy reduces redundant computation while preserving feature expressiveness, enabling the model to perform sufficient nonlinear transformation of high-dimensional features in pest images under lightweight constraints (Liu et al., 2022b).

Through the cascaded collaboration of SGFM and PCFN, HFFB accomplishes gating-enhanced reinforcement of spatially discriminative features and efficient nonlinear mapping of high-dimensional representations, while leveraging residual connections to ensure stable gradient propagation during deep network training. This design offers targeted advantages for the tobacco pest classification task: the spatial gating modulation of SGFM enables the model to adaptively focus on key discriminative regions such as wing venation textures and body color markings, while the partial convolution strategy of PCFN maintains sufficient expressive capacity for fine-grained morphological features while simultaneously constraining model complexity, thereby enhancing the practical utility of the model in resource-constrained field deployment scenarios.

### 3.4 Channel-spatial attention module

During feature extraction, CNNs inevitably capture redundant information from background clutter and noise, which can impair pest classification performance. Attention mechanisms can mitigate this issue by directing the network to focus on informative feature representations while suppressing irrelevant background and noise signals, thereby improving classification accuracy. Within each MSPP module, ECA is placed at the end of each of the four dilated-convolution sub-branches, followed by sequential ECA and SA refinement after the final MSPP feature fusion. An additional ECA–SA sequence is placed after the 1 × 1 convolution and BN in the feature-enhancement module.

The structure of the ECA module is illustrated in **Figure 6**. Compared to the SE module (Hu et al., 2018), which relies on two fully connected layers for channel recalibration, ECA (Wang et al., 2020b) introduces a lightweight one-dimensional convolution to model local cross-channel interactions, substantially reducing parameter count while preserving channel attention modeling capability. Given its high efficiency and low computational cost, the ECA module is integrated into the proposed network. Specifically, ECA first applies global average pooling to the input feature map to compress spatial information into a channel descriptor vector. The resulting vector is then reshaped and transposed before being fed into a one-dimensional convolutional layer with kernel size k, which captures local interaction information between adjacent channels. The kernel size k is adaptively determined based on the number of channels C, as formulated in Equation 9:

**Figure 6:**
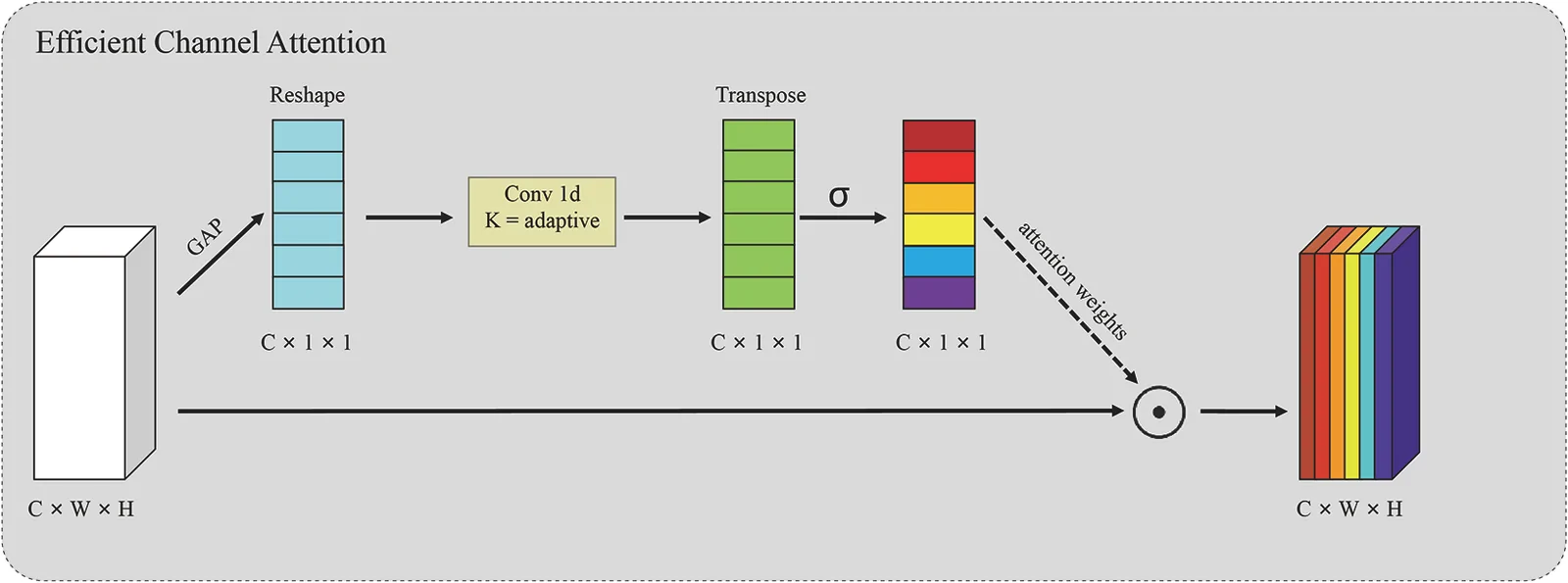
Structure of ECA.

(9) $$k=max(|\frac{\log_{2}C+1}{2}{|}_{\text{odd}},3)$$

where $|\cdot {|}_{\text{odd}}$ denotes rounding to the nearest odd number. Following the one-dimensional convolution, a Sigmoid activation function is applied to generate the channel attention weights, which are then multiplied channel-wise with the input feature map to complete channel-wise feature recalibration.

The structure of the SA module is illustrated in **Figure 7**. Unlike the standard CBAM (Woo et al., 2018), which employs a two-pathway spatial descriptor strategy using only global average pooling and global max pooling, the SA module in this study introduces a four-pathway spatial descriptor strategy. Specifically, the global channel average, global channel maximum, local 3×3 average pooling response, and local 3×3 max pooling response are concatenated along the channel dimension to construct a four-channel spatial descriptor map. Compared to the two-pathway approach, the four-pathway strategy is capable of simultaneously capturing both global and local spatial saliency information, making it more effective for localizing small-bodied or partially occluded pest targets. The resulting descriptor map is subsequently processed through a 7×7 convolutional layer, BN, and Sigmoid activation to generate the spatial attention weights. The core computation is formulated as Equation 10:

**Figure 7:**
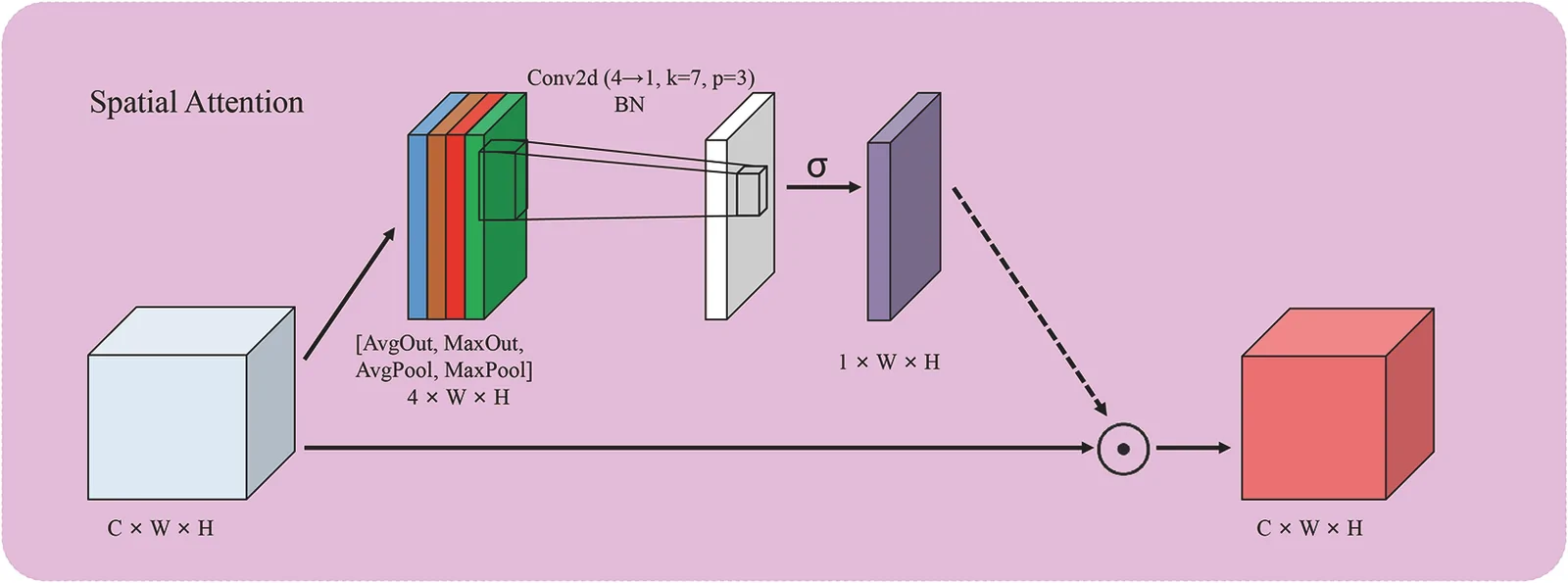
Structure of SA.

(10) $$M_{s}(F)=\sigma (Conv_{7\times 7}(Concat[F_{avɡ}^{s},F_{\text{max}}^{s},F_{avɡpool}^{s},F_{maxpool}^{s}]))$$

where $F_{avɡ}^{s}$ and $F_{\text{max}}^{s}$ denote the global channel average and maximum statistics maps, respectively, $F_{avɡpool}^{s}$ and $F_{maxpool}^{s}$ denote the local 3×3 average and max pooling response maps, respectively, and $Conv_{7\times 7}$ denotes the 7×7 convolutional operation. Following dual attention refinement by the ECA and SA modules, the network is able to selectively identify the most discriminative feature responses for the tobacco-associated categories along the channel dimension, while simultaneously concentrating spatial attention on the target-organism regions. This effectively suppresses redundant information introduced by background interference, providing high-quality feature representations for the classification head.

## 4 Experimental results and analysis

### 4.1 Experiment details

Model training and the primary classification experiments were implemented in Python 3.9.0 with PyTorch 1.12.1 under Windows 10. The hardware platform comprised a 12th-generation Intel Core i5-12600KF CPU and an NVIDIA GeForce RTX 4060 Ti GPU with 16 GB of VRAM. To address class imbalance, Focal Loss (Lin et al., 2017) was used with α = 0.25, γ = 1.5, and label smoothing of 0.05. The remaining training hyperparameters, including the optimizer, learning rate, batch size, and number of epochs, are summarized in **Table 4**.

**Table 4:** Hyperparameter configuration for model training.

Parameter	Value
Optimizer	AdamW
Loss Function	Focal Loss (α=0.25, γ=1.5)
Training Epochs	120
Warmup Epochs	5
Initial Learning Rate	8e-4
Learning Rate Scheduler	Cosine Annealing
Batch size	32
Weight Decay	2e-4

Except for the transfer-learning baselines reported in **Table 5**, all models in this study were trained from random initialization. For the transfer-learning comparison, MobileNetV3, EfficientNet-B0, DenseNet121, and ViT-Base were initialized with ImageNet-1K pretrained weights. Their classification layers were replaced for the 13-class task, and no backbone layers were frozen during fine-tuning. The pretrained models were trained using the same dataset partition and data augmentation procedure for up to 120 epochs with AdamW, a batch size of 32, an initial learning rate of $8\times 10^{-4}$, a weight decay of $2\times 10^{-4}$, five warm-up epochs, and cosine-annealing learning-rate scheduling.

**Table 5:** Comparison of MSFANet trained from scratch and ImageNet-pretrained models on the test set.

Model	Initialization	Accuracy	Precision	Recall	F1	Param(M)
MSFANet	Random	0.9763	0.9789	0.9759	0.9771	1.21
MobileNetV3	ImageNet-1K	0.9845	0.9869	0.9843	0.9855	1.53
EfficientNet-B0	ImageNet-1K	0.9818	0.9838	0.9814	0.9825	4.02
DenseNet121	ImageNet-1K	0.9872	0.9887	0.9874	0.9879	6.97
ViT-Base	ImageNet-1K	0.9463	0.9486	0.9480	0.9468	85.81

MSFANet was trained from random initialization, whereas the comparison models were initialized with ImageNet-1K pretrained weights. Their classification layers were replaced for the 13-class task, and no backbone layers were frozen during fine-tuning.

Model classification performance was comprehensively evaluated using four metrics: accuracy (Acc), precision (P), recall (R), and F1-score (F1). Accuracy measures the proportion of all samples correctly classified; precision measures the proportion of samples predicted as belonging to a given category that truly belong to that category; recall measures the proportion of samples that truly belong to a given category that are correctly identified as such; and F1 represents the harmonic mean of precision and recall, providing an overall measure of classification performance. The computational formulas for each metric are given in Equations 11–14:

(11) $$Acc=\frac{TP+TN}{TP+TN+FP+FN}$$

(12) $$P=\frac{TP}{TP+FP}$$

(13) $$R=\frac{TP}{TP+FN}$$

(14) $$F1=\frac{P\times R}{P+R}\times 2$$

where TP denotes the number of samples truly belonging to a given category and correctly predicted as such; FP denotes the number of samples that do not truly belong to a given category but are incorrectly predicted as belonging to it; FN denotes the number of samples truly belonging to a given category but incorrectly predicted as belonging to another category; and TN denotes the number of samples that do not truly belong to a given category and are correctly predicted as not belonging to it.

### 4.2 Performance of MSFANet

The loss curves and accuracy curves of MSFANet on the training and validation sets are presented in **Figure 8**. As illustrated, both the training loss and validation loss exhibit a consistent downward trend with increasing training epochs, while the accuracy curve rises steadily and converges after approximately 40 epochs. The training and validation curves followed similar trends, with no marked divergence over the observed epochs, indicating stable optimization under the adopted data split.

**Figure 8:**
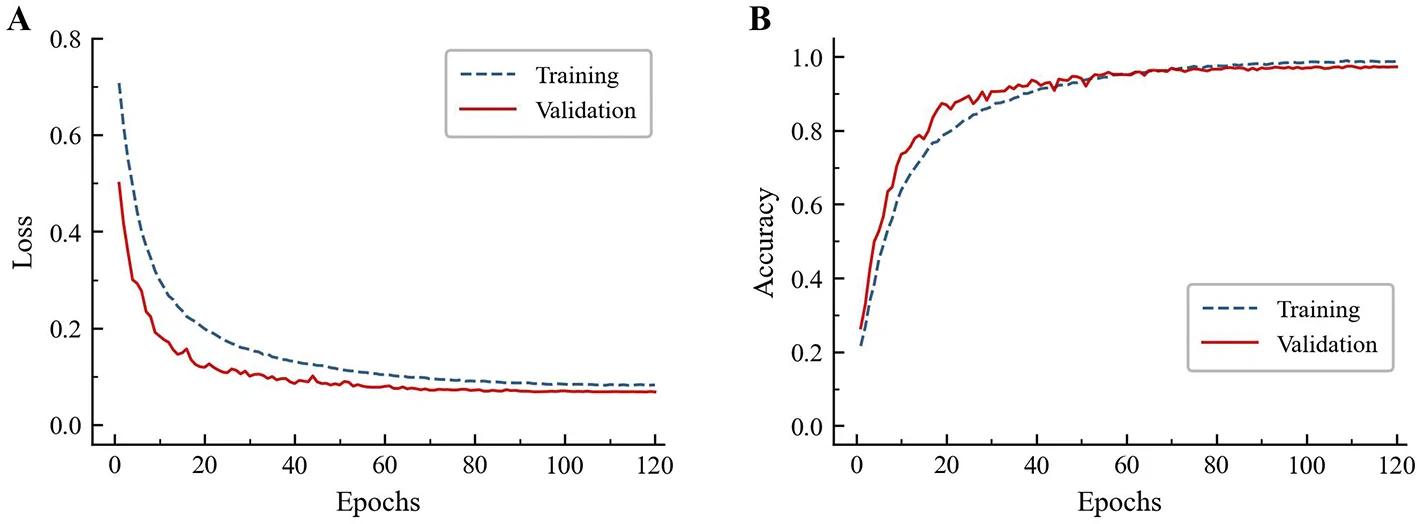
Training details of the model. **(A)** Training loss and validation loss of the model. **(B)** Training accuracy and validation accuracy of the model.

MSFANet was evaluated on the test set and achieved an accuracy of 97.63%, a macro-precision of 97.89%, a macro-recall of 97.59%, and a macro-F1 score of 97.71%, with a test loss of 0.0659. These results indicate stable classification performance across the 13 tobacco-associated categories. **Figure 9** presents the confusion matrix, in which the vertical and horizontal axes represent the true and predicted categories, respectively. The predominantly diagonal distribution indicates that most test images were correctly classified. Tobacco budworm showed a relatively lower classification accuracy than the other categories, which may be associated with its morphological and chromatic similarity to the larval stages of other pests. Overall, the results indicate strong test-set performance across the 13 tobacco-associated categories.

**Figure 9:**
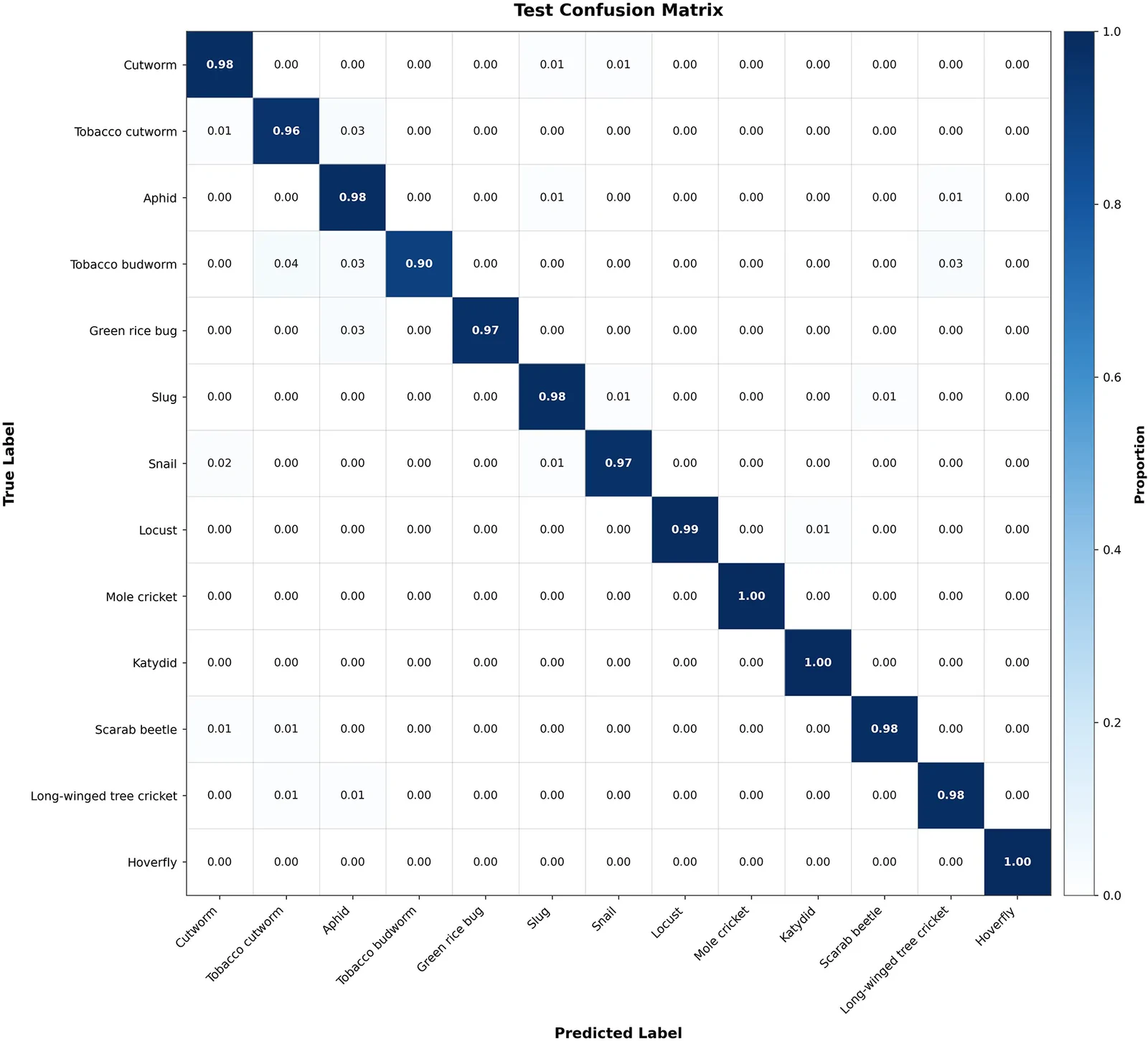
Confusion matrix for the 13 tobacco-associated categories on the test set.

Despite the strong overall classification performance, a small number of test images remained misclassified. As shown in **Figure 10**, four representative cases were selected to characterize these residual errors. Confusion between tobacco budworm and tobacco cutworm was associated with similar larval body profiles and longitudinal markings. The aphid sample provided limited discriminative information because the target occupied a small image region and exhibited low contrast against the leaf surface. In the slug and cutworm samples, coarse body contours and textures were more visually prominent than category-specific diagnostic features, particularly against soil backgrounds or in curled postures. These examples suggest that residual errors mainly occurred in visually ambiguous cases involving fine-grained inter-class resemblance, small effective target size, or reduced target–background contrast.

**Figure 10:**
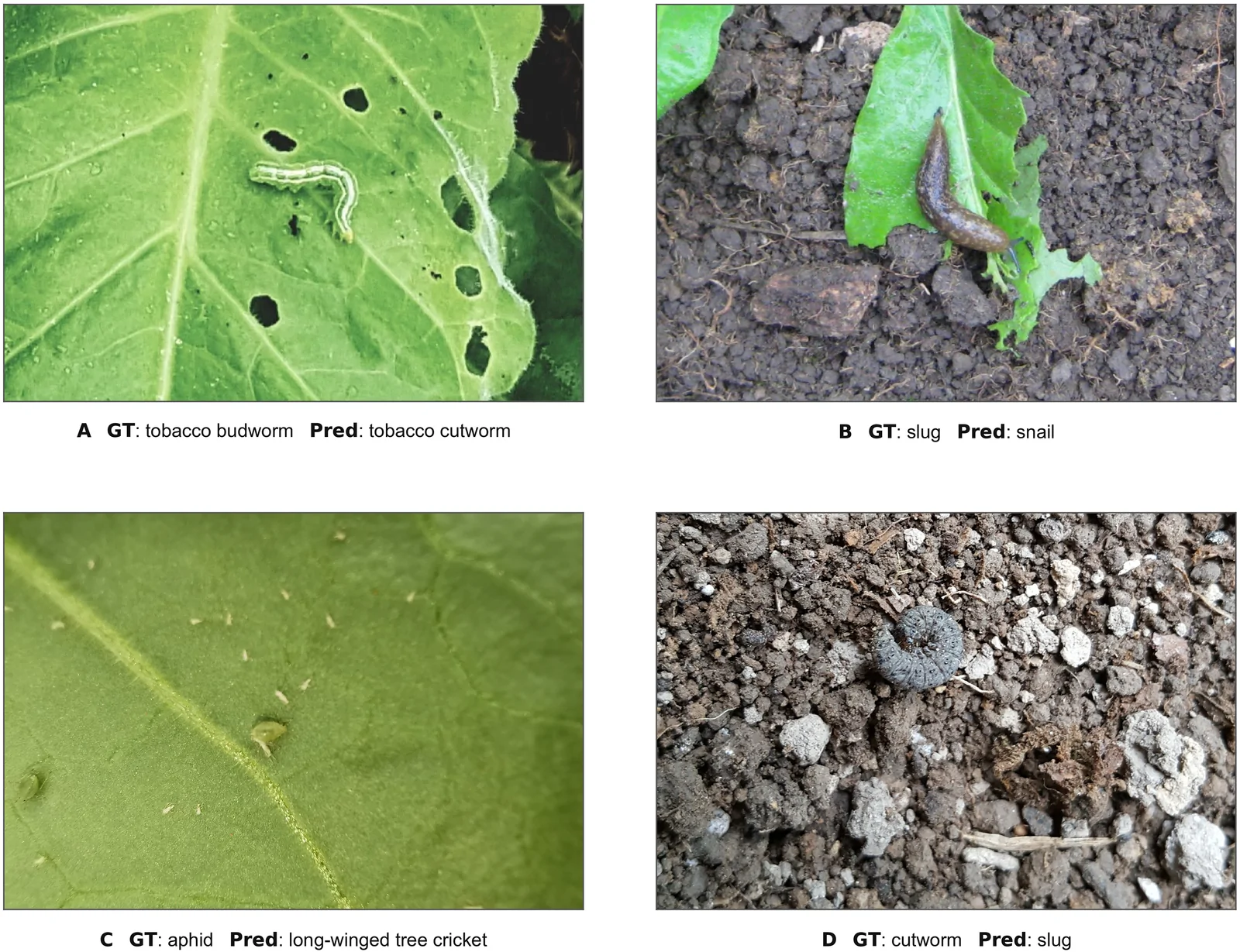
Representative misclassification cases on the test set. GT, ground truth; Pred, model prediction. **(A)** Tobacco budworm misclassified as tobacco cutworm. **(B)** Slug misclassified as snail. **(C)** Aphid misclassified as long-winged tree cricket. **(D)** Cutworm misclassified as slug.

### 4.3 Comparison with different models

To evaluate the classification performance and model compactness of MSFANet, comparative experiments were conducted using representative CNN, Transformer-, and lightweight architectures. The original comparison models included ResNet50, DenseNet121, EfficientNet-B0, MobileNetV2, MobileNetV3, ShuffleNetV2, ConvNeXt-Base, and ViT-Base. In response to recent advances in mobile-oriented network design, four additional lightweight architectures were evaluated: FasterNet-T0 (Chen et al., 2023), StarNet-S150 (Ma et al., 2024), RepViT-M0.9 (Wang et al., 2024a), and MobileNetV4 (Qin et al., 2024). All models reported in **Table 6** were trained from random initialization and evaluated using the same dataset partition, an input resolution of 224 × 224 pixels, data augmentation strategy, training protocol, and evaluation metrics. The ImageNet-pretrained models were evaluated separately under the transfer-learning protocol described in Section 4.1.

**Table 6:** Performance and parameter comparison of models trained from random initialization on the test set.

Model	Accuracy	Precision	Recall	F1	Param (M)
ConvNeXt-Base	0.9572	0.9588	0.9590	0.9588	87.58
EfficientNet-B0	0.9636	0.9675	0.9619	0.9642	4.02
MobileNetV2	0.9554	0.9592	0.9548	0.9566	2.24
MobileNetV3	0.9590	0.9635	0.9592	0.9608	1.53
ResNet50	0.9727	0.9743	0.9704	0.9720	23.53
ShuffleNetV2	0.9608	0.9662	0.9612	0.9635	1.27
DenseNet121	**0.9763**	**0.9792**	0.9757	**0.9772**	6.97
ViT-Base	0.9208	0.9162	0.9170	0.9163	85.81
FasterNet-T0	0.9563	0.9603	0.9573	0.9584	2.64
StarNet-S150	0.9563	0.9606	0.9573	0.9584	1.37
RepViT-M0.9	0.9681	0.9686	0.9689	0.9685	4.72
MobileNetV4	0.9508	0.9550	0.9514	0.9524	2.51
**MSFANet**	**0.9763**	0.9789	**0.9759**	0.9771	**1.21**

Precision, recall, and F1 are unweighted macro-averages across the 13 classes; macro-F1 was obtained by averaging the class-wise F1 scores rather than from macro-precision and macro-recall. Values were rounded to four decimal places after aggregation. Bold values indicate the highest value, whereas underlined values indicate the second-highest value for each metric.

**Figure 11** presents the validation accuracy curves of the models trained from random initialization over 120 training epochs. Differences in early-stage convergence were observed among the comparison models, whereas most models gradually entered a stable region during the later training stage. MSFANet showed a steady improvement in validation accuracy and remained within the upper-performing group after approximately 50 epochs. As shown in the enlarged region from epochs 80 to 120, its validation accuracy was generally maintained above 96%, with relatively limited epoch-to-epoch fluctuations. These results indicate that MSFANet achieved stable convergence under the adopted training protocol.

**Figure 11:**
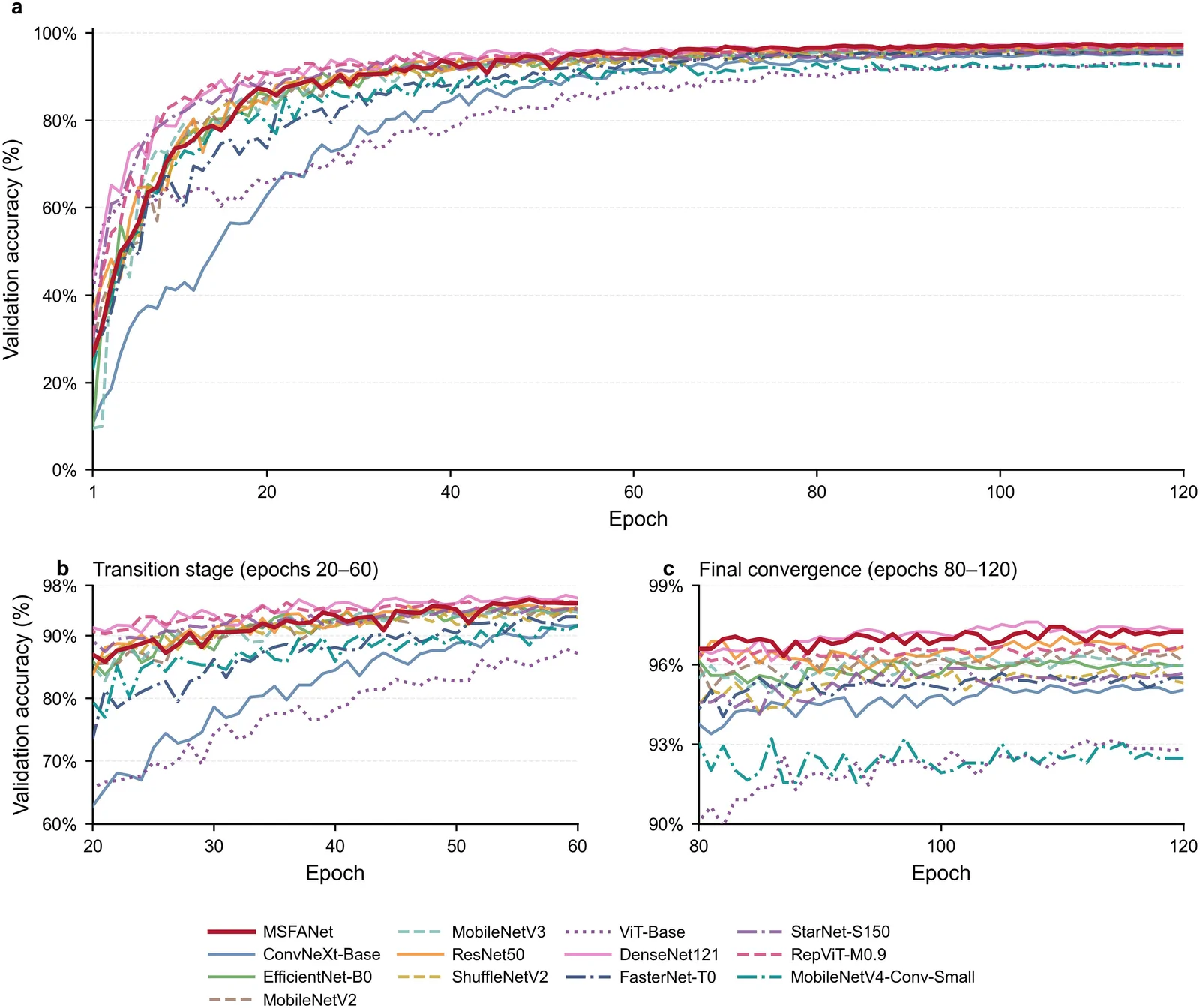
Validation accuracy curves of MSFANet and the comparison models over 120 training epochs. **(a)** Complete training trajectories; **(b)** enlarged view of the transition stage from epochs 20 to 60; and **(c)** enlarged view of the final convergence stage from epochs 80 to 120.

The test-set results are summarized in **Table 6**. Among the four newly added lightweight architectures, RepViT-M0.9 achieved the highest accuracy and macro-F1 score, reaching 0.9681 and 0.9685, respectively, with 4.72 M parameters. FasterNet-T0 and StarNet-S150 both achieved an accuracy of 0.9563, whereas MobileNetV4 achieved an accuracy of 0.9508. MSFANet achieved an accuracy of 0.9763 and a macro-F1 score of 0.9771 with 1.21 M parameters. Compared with RepViT-M0.9, MSFANet improved accuracy and macro-F1 by 0.82 and 0.86 percentage points, respectively, while reducing the parameter count by approximately 74.4%.

Across all evaluated models, MSFANet and DenseNet121 achieved the same highest accuracy of 0.9763, while their macro-F1 scores were nearly identical (0.9771 and 0.9772, respectively). However, MSFANet required only 1.21 M parameters, compared with 6.97 M for DenseNet121, corresponding to an approximately 82.6% reduction. MSFANet also had the lowest parameter count among all models included in the comparison. These results show that MSFANet maintained competitive classification performance while providing a favorable balance between predictive performance and model compactness.

A separate transfer-learning comparison is reported in **Table 5**. ImageNet-pretrained DenseNet121 achieved the highest accuracy of 0.9872, while pretrained MobileNetV3 and EfficientNet-B0 also achieved higher accuracy than MSFANet. In contrast, ViT-Base achieved a lower accuracy of 0.9463. MSFANet nevertheless achieved an accuracy of 0.9763 without external pretraining and retained the lowest parameter count of 1.21 M. Because the models were evaluated under different initialization and training regimes, these results are presented as a complementary transfer-learning benchmark rather than a controlled architectural comparison.

### 4.4 Ablation studies

To isolate the contributions of the architectural components and training strategies, two sets of controlled ablation experiments were conducted under the same data split, preprocessing pipeline, and optimization settings (**Table 7**). Panel A evaluates the architectural components. In the strict baseline, the MSPP and HFFB operations in all five MSFA blocks were replaced with identity mappings, without introducing surrogate convolutional layers. The remaining network and training strategy, including Focal Loss and the auxiliary classification head, were unchanged. Panel B retains the complete MSFANet architecture while varying the loss function and auxiliary classification head. Cross-entropy loss used the same label-smoothing factor of 0.05 as Focal Loss.

**Table 7:** Ablation results for the architectural components and training strategies of MSFANet.

A. Architectural component ablation								
MSPP	HFFB	Loss	Aux.	Accuracy	Precision	Recall	F1	Param (M)
–	–	Focal	✓	0.9481	0.9529	0.9489	0.9501	0.202
✓	–	Focal	✓	0.9718	0.9722	0.9720	0.9719	0.838
–	✓	Focal	✓	0.9709	0.9736	0.9707	0.9718	0.578
✓	✓	Focal	✓	0.9763	0.9789	0.9759	0.9771	1.213
B. Training-strategy ablation								
✓	✓	CE	–	0.9681	0.9702	0.9683	0.9691	1.212
✓	✓	CE	✓	0.9663	0.9719	0.9668	0.9690	1.213
✓	✓	Focal	–	0.9709	0.9711	0.9704	0.9704	1.212
✓	✓	Focal	✓	0.9763	0.9789	0.9759	0.9771	1.213

In Panel A, Focal Loss and the auxiliary classification head were retained for all variants. In Panel B, the complete MSFANet architecture was retained. The configuration containing MSPP, HFFB, Focal Loss, and the auxiliary classification head represents the full MSFANet and is repeated as the common reference in both panels. CE denotes cross-entropy loss.

As shown in **Table 7**, Panel A, the strict baseline achieved an accuracy of 94.81% and a macro-F1 score of 95.01% with 0.202 M parameters. Introducing MSPP alone increased the accuracy and macro-F1 score to 97.18% and 97.19%, respectively. HFFB alone achieved corresponding values of 97.09% and 97.18%. The comparable improvements obtained by the two single-module variants are consistent with their intended but distinct functions. MSPP aggregates contextual information across multiple receptive fields, whereas HFFB refines feature representations through spatial gating and partial convolution. The complete MSFANet achieved the highest accuracy of 97.63% and macro-F1 score of 97.71%. It exceeded the MSPP-only variant by 0.45 and 0.52 percentage points and the HFFB-only variant by 0.55 and 0.53 percentage points, respectively. These results indicate that both modules contributed to the final performance and that retaining them jointly was more effective than using either module alone.

Panel B evaluates the effects of Focal Loss and the auxiliary classification head. Without auxiliary supervision, replacing cross-entropy loss with Focal Loss increased the accuracy from 96.81% to 97.09% and the macro-F1 score from 96.91% to 97.04%. Under Focal Loss, introducing the auxiliary head further increased the accuracy to 97.63% and the macro-F1 score to 97.71%. In contrast, the auxiliary head did not improve accuracy or macro-F1 when combined with cross-entropy loss. The effect of auxiliary supervision therefore depended on the loss formulation rather than being uniform across all training settings. Among the evaluated configurations, the combination of Focal Loss and auxiliary supervision achieved the best overall performance.

Because green rice bug was the least represented category, with 36 images in the test set, its classification results were examined separately. All four training configurations correctly classified 35 of the 36 green rice bug images, corresponding to a recall of 97.22%. Both cross-entropy configurations and the complete MSFANet achieved a precision of 100% and an F1 score of 98.59%. Focal Loss without auxiliary supervision produced a precision of 94.59% and an F1 score of 95.89% because two samples from other categories were incorrectly predicted as green rice bug. Thus, Focal Loss alone did not provide a consistent class-specific advantage for green rice bug, although it produced modest improvements in the overall macro-averaged metrics.

### 4.5 Visualization analysis and feature interpretability

To further interpret the discriminative basis of each model, Grad-CAM (Selvaraju et al., 2017) was employed to visualize the feature activation regions of different models. ResNet50, MobileNetV3, and ViT-Base were selected as representative comparison models, and test samples were chosen to cover typical scenarios including simple backgrounds, complex backgrounds, and visually similar categories. The resulting activation maps are presented in **Figure 12**. The heatmaps of ResNet50 and MobileNetV3 exhibit broadly diffuse high-activation regions with dispersed energy distributions and noticeable background interference responses, indicating inherent limitations in feature focusing for both models. Although ViT-Base possesses global modeling capability, its activation regions exhibit fragmented and discrete distributions, with particularly unstable responses to small pest targets, suggesting insufficient capacity for fine-grained local feature capture. In contrast, the activation heatmaps of MSFANet consistently concentrate high-response regions precisely on the pest body across all test samples. Compared with the selected baselines, the MSFANet heatmaps were more concentrated on visible pest regions in these examples, consistent with more localized feature responses. Nevertheless, Grad-CAM provides qualitative rather than quantitative evidence.

**Figure 12:**
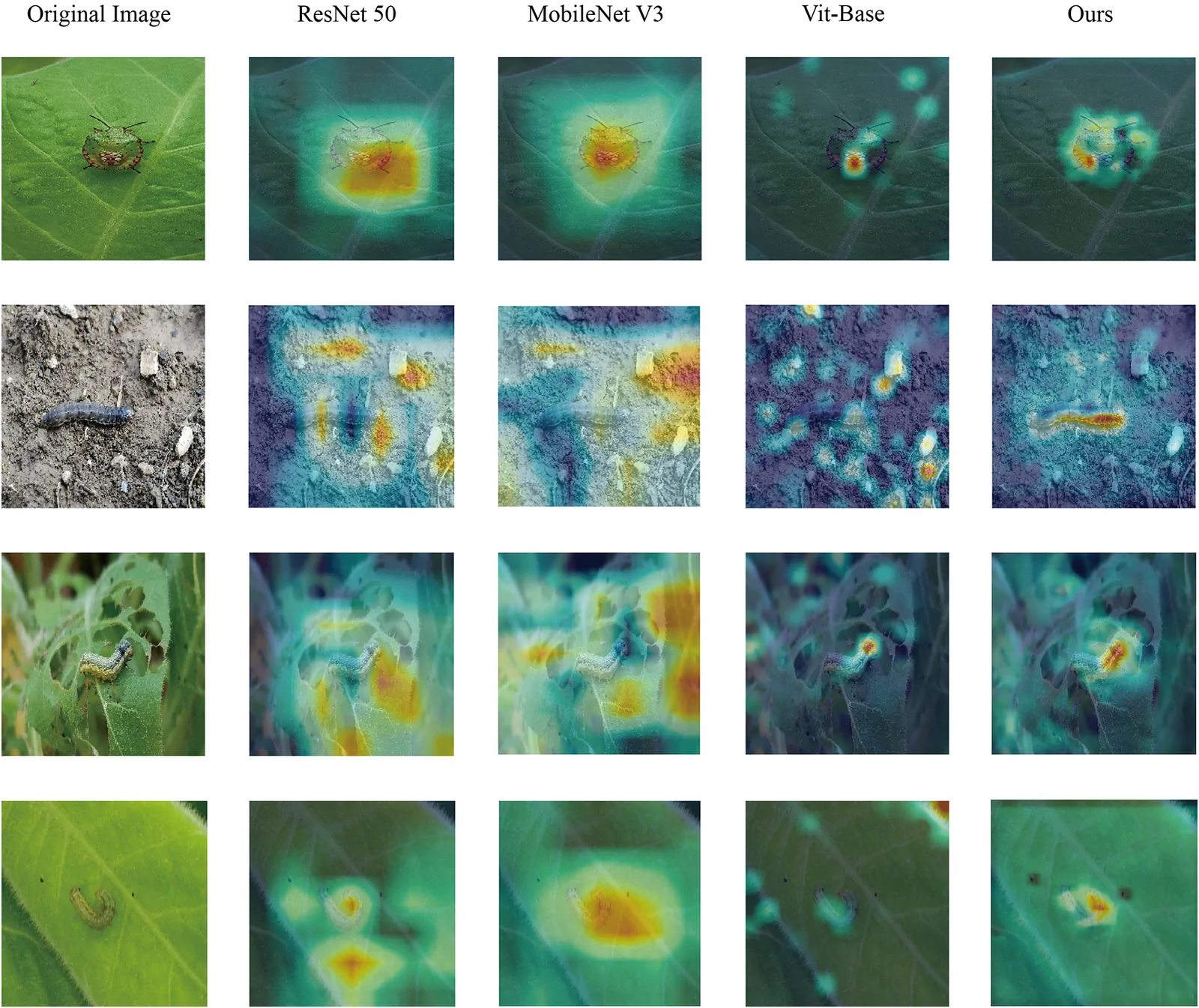
Grad-CAM visualizations for MSFANet and the comparison models. Red regions indicate higher model responses.

Furthermore, t-SNE dimensionality reduction (Zhao et al., 2022) was applied to the test-set features to qualitatively examine intra-class compactness and inter-class separability. As shown in **Figure 13**, most categories formed relatively compact clusters with limited overlap. Tobacco budworm and tobacco cutworm remained relatively close in the two-dimensional projection, consistent with their morphological similarity and the confusion observed for tobacco budworm in **Figure 9**. Because t-SNE is a nonlinear visualization method, these patterns are interpreted qualitatively rather than as quantitative evidence of class separability.

**Figure 13:**
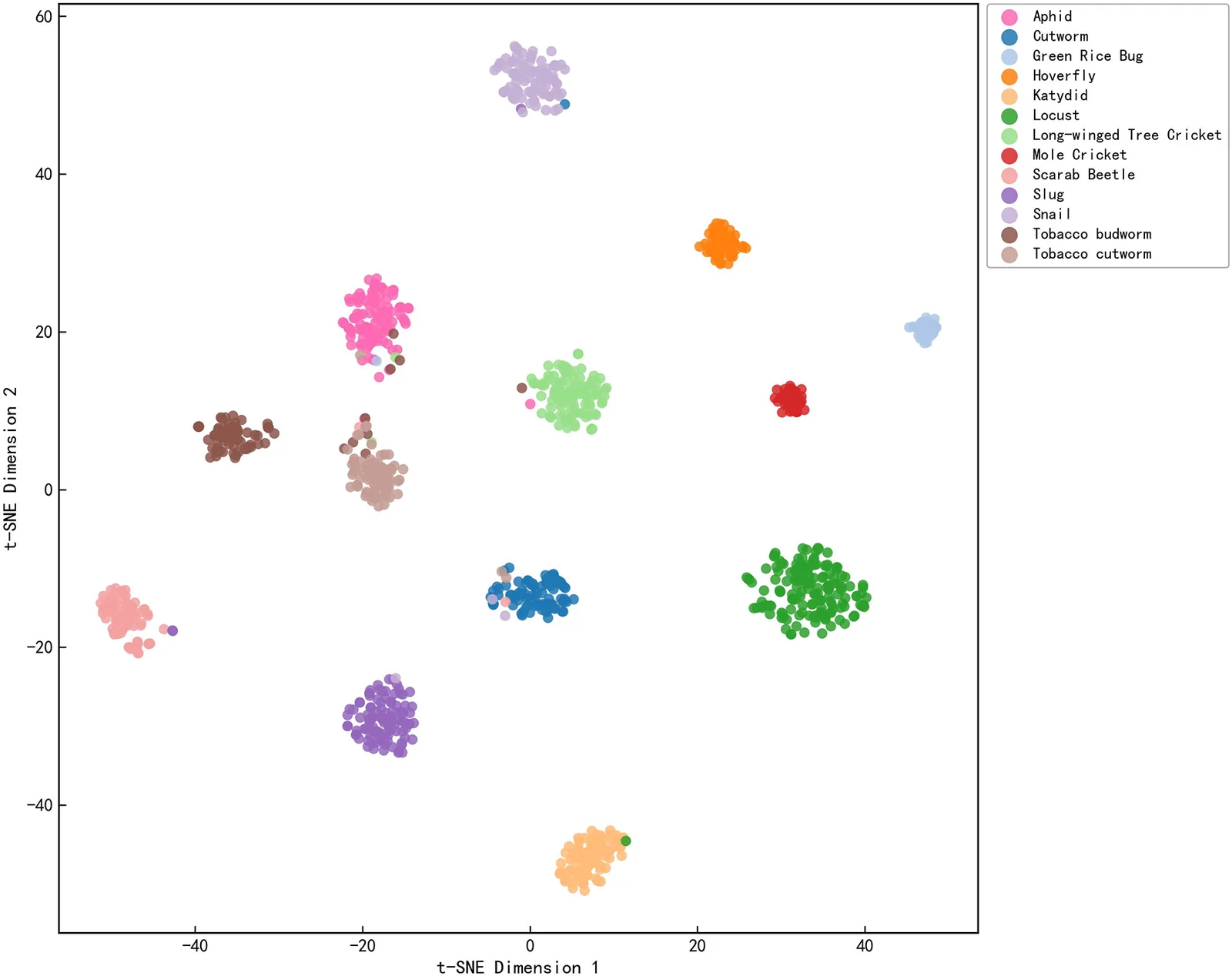
t-SNE visualization of MSFANet feature distributions on the test set.

Based on the above results, MSFANet, through the multi-scale feature aggregation of the MSPP module and the hierarchical feature refinement of the HFFB module, is capable of forming clearly separable category structures in the feature space, providing discriminative feature representations for classification across the 13 tobacco-associated categories.

### 4.6 PC-based deployment benchmark and dynamic quantization analysis

To assess deployment-oriented inference under controlled CPU conditions, MSFANet, MobileNetV2, MobileNetV3, and ShuffleNetV2 were independently evaluated in PyTorch FP32, ONNX FP32, and partially quantized ONNX formats on the same 1, 098-image test set. The experiment used PyTorch 2.2.2, ONNX 1.19.1, ONNX Runtime 1.19.2 with CPUExecutionProvider, and ONNX opset 13. Inference-only latency was measured on an Intel Core i5-12600KF x86 CPU using 224 × 224 inputs, batch size 1, and one CPU thread. Dynamic quantization was performed using onnxruntime.quantization.quantize_dynamic with per-tensor signed QInt8 weights, targeting MatMul/Gemm operators. Graph inspection identified two DynamicQuantizeLinear and two MatMulInteger operators in MSFANet and MobileNetV3, and one of each in MobileNetV2 and ShuffleNetV2. No convolution operators were quantized; hence, this procedure represents partial dynamic QInt8 quantization rather than full-network INT8 quantization.

As shown in **Table 8**, MSFANet retained the highest accuracy but showed higher latency than the three lightweight baselines. ONNX export reduced its mean latency from 446.12 to 276.71 ms, while partial dynamic quantization further reduced it to 190.11 ms. Although this latency remained higher than those of the three lightweight baselines, the sub-second inference time suggests potential for photo-based, single-image pest identification, where continuous video-rate processing is not required. All three runtime formats produced identical top-1 predictions for each model in the deployment benchmark. Accordingly, accuracy and the independently recalculated macro-precision, macro-recall, and macro-F1 remained unchanged. Relative to ONNX FP32, the quantized models produced zero changed predictions and 100% agreement. Nevertheless, their outputs were not numerically identical. The mean/maximum absolute logit deviations were 0.00660/0.07131 for MSFANet, 0.00944/0.05575 for MobileNetV2, 0.00400/0.03485 for MobileNetV3, and 0.00896/0.08979 for ShuffleNetV2. These results confirm independent execution while explaining the unchanged classification metrics.

**Table 8:** Deployment-oriented inference performance under the PC-based x86 CPU setting.

Model	Runtime format	File size (MiB)	Accuracy	Latency (ms)
MSFANet	PyTorch FP32	5.025	0.9763	446.12 ± 123.02
	ONNX FP32	4.950	0.9763	276.71 ± 6.00
	ONNX dynamic QInt8	5.050	0.9763	190.11 ± 79.07
MobileNet V2	PyTorch FP32	8.782	0.9554	45.28 ± 4.44
	ONNX FP32	8.524	0.9554	15.05 ± 1.68
	ONNX dynamic QInt8	8.491	0.9554	9.29 ± 4.48
MobileNet V3	PyTorch FP32	5.970	0.9599	10.16 ± 1.10
	ONNX FP32	5.858	0.9599	2.24 ± 0.28
	ONNX dynamic QInt8	4.147	0.9599	2.18 ± 0.27
ShuffleNet V2	PyTorch FP32	5.002	0.9608	11.69 ± 0.91
	ONNX FP32	4.869	0.9608	3.56 ± 0.40
	ONNX dynamic QInt8	4.857	0.9608	3.59 ± 0.55

Latency is reported as the mean ± standard deviation from three technical timing passes over the 1, 098-image test set, corresponding to 3, 294 inference-only observations per runtime format after 20 warm-up runs. Measurements were performed using batch size 1 and one CPU thread, excluding image loading and preprocessing. PyTorch file sizes correspond to weights-only state dictionaries and do not include optimizer states or other training metadata. Dynamic QInt8 denotes partial dynamic quantization of MatMul/Gemm operations; convolution operations remained in FP32.

Partial quantization affected model storage and latency differently across these convolution-dominated networks. For MSFANet, the model size increased slightly from 4.950 to 5.050 MiB after quantization. Only two classifier-side matrix multiplications were quantized, whereas 167 convolution operators remained in FP32. The limited reduction in quantized weights, together with the additional quantization nodes, likely explains the small increase in file size. The MobileNetV3 CPU evaluation correctly classified one more image than the original GPU mixed-precision evaluation in **Table 6**, although the checkpoint, dataset, model architecture, class mapping, and preprocessing were identical. **Table 8** therefore reports the independently measured CPU result. Finally, these measurements were obtained on a desktop x86 CPU and do not represent ARM-based or on-device mobile performance. Evaluation of device-specific latency, memory consumption, and energy use on physical mobile hardware remains necessary.

## 5 Discussion

The performance gains of MSFANet are consistent with distinct contributions from MSPP and HFFB. MSPP aggregates contextual information across multiple receptive fields through parallel dilated convolution and multi-scale pooling branches. Such operations can expand the effective receptive field and improve the representation of objects with substantial scale variation (Chen et al., 2018; Qian et al., 2025; Deng et al., 2025). HFFB further refines feature representations through spatial gating and partial convolution, which may help suppress irrelevant background responses and preserve discriminative pest features. The concentrated activation regions observed in the Grad-CAM visualizations are consistent with this intended refinement function. Importantly, both single-module variants substantially outperformed the strict baseline, while the complete configuration achieved the highest overall performance. The results therefore support contributions from both modules but do not indicate that HFFB is effective only in the presence of MSPP. Because the two single-module variants achieved similar results, no claim is made that either module contributed more strongly than the other.

The training-strategy ablation provides a more qualified assessment of Focal Loss and auxiliary supervision. Focal Loss produced modest improvements in the overall macro-averaged metrics when compared with cross-entropy loss without auxiliary supervision. However, the auxiliary head did not improve accuracy or macro-F1 under cross-entropy loss, whereas its combination with Focal Loss achieved the best overall results. Moreover, the identical green rice bug recall across all training settings indicates that the overall improvement cannot be interpreted as a uniform gain for every minority category. These findings support the selected training configuration for overall classification performance while also defining the limits of its class-specific benefit.

Multi-scale feature extraction has emerged as an important research direction in the field of agricultural pest recognition. Wang et al. (2024b) extracted multi-scale features using a dilated Inception module combined with an attention mechanism, achieving a detection accuracy of 92.16% on the IP102 dataset. Peng et al. (2023) introduced Selective Kernel units to dynamically adjust receptive fields, attaining a classification accuracy of 75.28% on the HQIP102 dataset. Qian et al. (2025) integrated multi-scale feature fusion with a hybrid attention mechanism and incorporated a Transformer module to model global contextual dependencies, achieving a classification accuracy of 75.74% on the IP102 dataset. Lv et al. (2024) constructed a self-built rice pest and disease dataset and proposed a Lightweight Multi-scale Feature Extraction Network, achieving an average classification accuracy of 95.38% with only 1.4 M parameters. Suzauddola et al. (2025) proposed CMPestNet, validated on the self-constructed jute pest dataset Jute17 and the Pest24 dataset, where its Generalized Multi-Scale Feature Extraction module captures fine-grained cross-scale feature relationships, achieving an mAP of 78.22% on Pest24. Collectively, these studies demonstrate the value of multi-scale feature extraction across pest-related computer vision tasks. In comparison, MSFANet was developed for image-level classification across 13 tobacco-associated categories under field conditions. By integrating the multi-scale contextual aggregation of MSPP with the hierarchical feature refinement of HFFB, it achieved an accuracy of 97.63% on the 13-class task with only 1.21 M parameters.

The present study has three main limitations. First, the dataset remains class-imbalanced and was collected from specific tobacco-growing environments with limited temporal and climatic coverage. Generalization under underrepresented conditions, such as intense illumination, rain, and fog, therefore requires further validation. Second, the dataset contains multi-individual images and includes different developmental stages for several categories, but these attributes were not independently annotated. Their effects on classification performance could therefore not be evaluated separately according to developmental stage, instance count, overlap, or occlusion severity. Robustness in real-time video streams also remains unverified. Third, MSFANet is a closed-set, single-label image classifier. This formulation permits multiple individuals from the same category to share one image-level label, but it cannot localize or count individuals, distinguish co-occurring categories, or reject organisms outside the 13 trained categories. Anomaly detection or calibrated confidence thresholds are therefore needed to reject unfamiliar or uncertain inputs before practical deployment (Batz et al., 2023; Shen et al., 2024).

The deployment benchmark was conducted on a desktop x86 CPU and should not be interpreted as an ARM-device simulation. Runtime, memory use, and model compatibility may differ across ARM CPUs, embedded NPUs, and Jetson platforms because hardware backends support different operators. Future work will first enrich the dataset with stage- and instance-level annotations and more challenging field images or video sequences. MSFANet will then be extended to detection or segmentation frameworks, together with confidence calibration and open-set rejection. Convolution-aware static quantization, quantization-aware training, and structured pruning will also be investigated for platform-specific acceleration (Zhang and Lv, 2024). The optimized models will be evaluated on physical edge devices using latency, peak memory, energy consumption, and prediction stability as deployment criteria.

## 6 Conclusions

This study presents MSFANet, a multi-scale feature aggregation network for tobacco pest recognition, which is formulated as single-label image classification in this work. The network was designed to address substantial variation in pest scale, complex field backgrounds, and class imbalance. The core architecture of the proposed model combines two task-oriented modules: MSPP, which captures cross-scale spatial contextual features in images of tobacco-associated organisms through parallel feature extraction via dilated convolutional branches and multi-scale pooling branches, combined with a lightweight cross-attention fusion mechanism; and HFFB, which achieves efficient hierarchical feature refinement under lightweight constraints through the cascaded integration of SGFM and PCFN. MSFANet achieved a classification accuracy of 97.63% on the 13-class task with only 1.21 M parameters and without external pretraining, demonstrating a favorable balance between classification performance and model compactness. These findings support its potential use in automated monitoring of tobacco pests and natural enemies. The current dataset was collected from specific tobacco field environments, and both the number of tobacco-associated categories and the diversity of field scenarios remain limited, warranting further expansion. Future work will expand the dataset across more diverse field environments and investigate convolution-aware static quantization, quantization-aware training, and structured pruning. These approaches will subsequently be evaluated on physical ARM-based mobile and edge devices to characterize latency, memory consumption, energy efficiency, and classification stability under realistic deployment conditions.

## Data Availability

The original contributions presented in the study are included in the article/supplementary material. Further inquiries can be directed to the corresponding authors.
